# Prevalence of Occupational Asthma and Respiratory Symptoms in Foundry Workers

**DOI:** 10.1155/2013/370138

**Published:** 2013-09-22

**Authors:** Servet Kayhan, Umit Tutar, Halit Cinarka, Aziz Gumus, Nurhan Koksal

**Affiliations:** ^1^Department of Chest Disease, Faculty of Medicine, Recep Tayyip Erdogan University, 53100 Rize, Turkey; ^2^Department of Chest Disease, Hospital of Chest Disease and Thoracic Surgery, 55090 Samsun, Turkey; ^3^Department of Chest Disease, Faculty of Medicine, Ondokuz Mayis University, 55139 Samsun, Turkey

## Abstract

This cross-sectional study was conducted in a foundry factory to assess the prevalence of respiratory symptoms and occupational asthma in foundry workers. Physical examination, spirometric evaluation, chest radiograph, and a questionnaire related to respiratory symptoms were performed. Monitoring of peak expiratory flow rates, spirometric reversibility test, and high-resolution computed tomographies were performed for the participants having respiratory symptoms and/or impaired respiratory function test. A total of 347 participants including 286 workers from production department and 61 subjects who worked in nonproduction departments were enrolled in this study. It is found that phlegm (*n*: 71, 20.46%) and cough (*n*: 52, 14.98%) were the most frequent symptoms. The other symptoms were breathlessness (*n*: 28, 8.06%), chest tightness (*n*: 14, 4.03%), and wheezing (*n*: 7, 2.01%) . The prevalence of occupational asthma was found to be more frequent among the subjects who worked in the production department (*n*: 48, 16.78% ) than the other persons who worked in the nonproduction department (*n*: 3, 4.91%) by chi-square test (*P*: 0.001). To prevent hazardous respiratory effects of the foundry production, an early diagnosis of occupational asthma is very important. Cessation of cigarette smoking and using of protective masks during the working time should be encouraged.

## 1. Introduction

The prevalence of occupational diseases shows the quality of working conditions and health of working environment. Respiratory diseases are common entities in occupational industries, because the lungs are the route of entry for noxious particles and gases. These agents can be inhaled in the form of fibers or dusts. The development of occupational respiratory disease is dependent on several factors including the chemical nature and physical state of the inhaled substance, the size and concentration of the dust particles, the duration of exposure, and individual susceptibility [1]. Respiratory irritants represent a major cause of occupational obstructive airway diseases related to irritative agents causing occupational asthma.

Work-related or occupational asthma is defined as a chronic inflammatory disorder of the airways with recurrent episodes of respiratory symptoms such as coughing, wheezing, chest tightness, dyspnea, shortness of breath at rest, and reversible airflow limitations caused by a particular occupational environment. The foundry workers are potentially exposed to a number of noxious particles and gases including asbestos, silica, diphenylmethane diisocyanate, polycyclic aromatic hydrocarbons, benzene, and sulfuric acid mist and toxic metals including zinc, chromium, nickel, and cadmium [2]. They have a risk of having respiratory symptoms and lifelong chronic obstructive airway diseases including asthma, COPD, pneumoconiosis, and cancers [3–7].

This study was designed to evaluate the effects of the foundry production on respiratory health of workers.

## 2. Materials and Methods

### 2.1. Study Design, Study Population, and Definitions

This is a cross-sectional study, and it was conducted at one of the foundry factories localized in the industrial region of Samsun, Turkey. A total of 347 workers including 286 workers from production department who were exposed to dust and noxious gases and 61 subjects from the other departments were enrolled in the study. The study was approved by the Local Ethics Committee. The participants were informed about the aim of the study. All participants were assessed with a modified questionnaire adopted from the European Community Respiratory Health Survey (ECRHS) by face-to-face interviews [8]. The relationships between work department and using of protective masks and respiratory symptoms including cough, phlegm, wheezing, chest tightness, breathlessness, and smoking history (pack/year) were evaluated.

A physician of pulmonary disease examined all participants, and an experienced technical staff for measuring the respiratory function test performed the test in the factory. Standard posteroanterior chest X-rays were taken for all subjects. High-resolution computed tomographies (HRCT) were also obtained in cases presenting with respiratory symptoms and obstructive and restrictive disorders in respiratory function tests and for the subjects with abnormal chest X-ray findings.

In the diagnosis of occupational asthma, the internationally recommended criteria are used. Subjects with one of the asthma symptoms that lessen or disappear when the subject leaves the work environment, with variability in PEF > 20%, and did not have a previous history of asthma before their employment were considered as occupational asthma [9, 10].

### 2.2. Working Environment

The foundry based factory is located in Samsun Industrial Zone and has an annual casting capacity of 30.000 tons. The production program of the factory covers the design and manufacture of the pumping and piping equipment such as the centrifugal, mixed, and axial flow pumps for water; gate, butterfly, check and air valves, ductile iron pipe fittings, tapping valves, and fire hydrants.

### 2.3. Exposure Assessment

Foundry workers are classified into 5 categories for exposure assessments: (1) core making, (2) moulding, (3) melting and pouring, (4) fettling (cleaning castings), and (5) after processing groups. Some workers in this study population had worked in more than one department at their shifts, and they have not worked in the separate locations according to the job categories. Job area was classified as the longest-held job during their foundry work. Thus, the workers in production department have been exposed to similar hazards regardless of job categories. And individual exposure assessment could not be done in this study. Core makers are exposed to isocyanate, but the concentrations of isocyanate could not be measured in this study. The working environment with a dust concentration was measured in 16 different parts of the factory, and dust concentration was reported as below maximum allowable concentration (MAC <10 mg/m^3^) in 14 departments and higher than MAC level in two departments; those were core making department (10.122 mg/m^3^) and fettling (cleaning casting) department (10.448 mg/m^3^). Workers in furnace and fettling were classified into the high-exposure group. Average respirable dust concentration was 0.216 mg/m^3^ for the moulding group, 0.322 mg/m^3^ for the melting and pouring group, and 0.216 mg/m^3^ for after processing group. The workers in moulding, melting and pouring, and after processing departments were classified into low-exposure group. Job categories were mainly classified into two groups as production and non-production according to working area.

### 2.4. Pulmonary Function Tests

Pulmonary function tests of all subjects were performed using an MIR Spirolab-II vitalograph (Italy) device in a sitting position and in accordance with the test procedures recommended by the American Thoracic Society [11]. Spirometric tests were performed at least three times for each worker and the best values were accepted. Forced vital capacity (FVC), forced expiratory volume at one second (FEV_1_), FEV_1_/FVC, peak expiratory flow (PEF), and forced expiratory flow 25–75% (FEF_25−75_) were measured. All measurements were expressed as the percentage of predicted values. The workers were evaluated in terms of respiratory diseases according to consensus reports of GINA for asthma and GOLD for COPD. It was considered to be abnormal if the tested FVC and PEF values were found to be below 80% of predicted value or FEV_1_/FVC was found to be below 70%. Reversibility test and peak expiratory flow (PEF) meter (SpiroFlow, PEF meter, USA) followup were used to diagnose occupational asthma in subjects with respiratory symptoms and restrictive or obstructive spirometric disorder. In reversibility test, pulmonary function tests were repeated 15 minutes later from the first test inhalation of 400 *μ*g salbutamol. A 12% increase in FEV_1_ percent of predicted or an absolute volume of 200 mL increase in FEV_1_ was considered as positive. The subjects were trained to use PEF meter, PEF measurements were performed 4 times daily, PEF variability was calculated, and the values >20% were considered to be positive [10].

### 2.5. Statistical Analysis

All statistical analyses were performed by using SPSS 16 programme. Descriptive analysis of data expressed as mean ± standard deviation (SD), range and percentage, and a *P*-value of <0.05 was used as the level of statistical significance. Between-group comparisons of parametric variables were made by a Student's *t*-test, and chi-square test was used for nonparametric variables.

## 3. Results

A total of 347 participants including 286 workers from the department of foundry production with the mean age 33.57 ± 7.0 and 61 subjects with the mean age 37.55 ± 9.3 who worked in non-production departments were enrolled in the study. It is found that phlegm (*n*: 71, 20.46%) and cough (*n*: 52, 14.98%) were the most frequent symptoms among the workers. The other symptoms were breathlessness (*n*: 28, 8.06%), chest tightness (*n*: 14, 4.03%), and wheezing in (*n*: 7, 2.01%) persons. Cough and phlegm were found to be related to smoking habit (*P*: 0.029). The symptoms of cough, phlegm, breathlessness, and chest tightness were found to be more frequent in the workers of foundry production department as is shown in Table 1 (*P*: 0.023, *P*: 0.001, *P*: 0.048, and *P*: 0.054, resp.). The prevalence of occupational asthma was found to be more frequent among the subjects who worked in foundry production department (*n*: 48, 16.78%) than the other persons who worked in non-production department (*n*: 3, 4.91%) (*P*: 0.003) as shown in Table 2. The workers who used protective masks all the time had a lower prevalence rate of respiratory symptoms and occupational asthma than those not using them (*P*: 0.039 and *P*: 0.001 respectively) as it is shown in Table 3.

The reversibility test with the variability in mean PEF records (>20%) was found during working days in these 51 individuals. Diurnal PEF variability (>20%) was also found in most of these groups (*n*: 32, 62.7%).

We found that smoking also increased the risk of occupational asthma in foundry workers. It is found that smokers were more frequent among asthmatics (*P*: 0.021), and degree of smoking (pack/year) was higher than that of nonasthmatics (*P*: 0.037). We did not diagnose any cancer and pneumoconiosis at study time by chest X-ray and HRCT. The prevalence of occupational asthma was found to be increased in the workers who were exposed to high concentrations of respirable dust, and the results of pulmonary function tests (FEV_1_% of predicted) with occupational asthma prevalence according to dust exposure are shown in Table 4.

## 4. Discussion

Occupational asthma became the second prevalent occupational lung disease following pneumoconiosis. Occupational asthma has been reported to be associated with several occupation groups in the literature including automobile and furniture painters, textile workers, plastics manufacturers, hairdressers, food processors, paper factory workers, farm workers, welders, and chemical processors [8]. The foundry workers also have a risk of occupational asthma. Furthermore, it was previously reported that there are an increased number of lung cancer cases among foundry workers [12]. The prevalence of pneumoconiosis was reported as 3.7% in 950 foundry workers, and they were classified as stage 1/0 or more advanced according to the International Labor Organization (ILO) classification [13]. In the present study, we observed that there is an increase in occupational asthma in foundry workers, and we did not find any pneumoconiosis and lung cancer cases. But long-term followup is needed to analyze the risk of neoplastic disease and pneumoconiosis in foundry workers. Cigarette smoking adversely affects the lung function of the workers, and exposure to air contaminants in the foundry may also impair the lung function additively, and we found similar results in this study.

We used questionnaire and PEF monitoring as an alternative method to nonspecific bronchial provocation test to demonstrate airway hyperreactivity [11]. Nonspecific bronchial provocation test requires experienced staff and can be performed in specific centers [14, 15]. According to the fact that the most of our study population did not give their consent to bronchial provocation test, we could not use the nonspecific bronchial provocation test to diagnose occupational asthma in this study.

A reduction in FEV_1_/FVC and FEV_1_ is an indicator of obstructive abnormalities, and a reduction in FEF_25−75_ is an indicator of small airway obstruction [16]. In a controlled study involving 166 workers exposed to chemicals in a paper factory, spirometric results (FEV_1_%, FVC%, FEF_25−75_%, and FEV_1_/FVC) were found to be lower in the workers compared to controls [17]. In another study involving the workers exposed to chemicals in a paper production factory, PFT was monitored for 3 years in certain intervals and the reductions in FEV_1_ and FVC were associated with the duration of employment [18].

Limitation of the present study is the fact that it is a cross-sectional study, and long-term followup results of the foundry workers are not studied. We used only the respirable dust concentrations for exposure analysis. Foundry workers are exposed to some other chemicals and gases such as isocyanates. But individual dust and gas exposure assessment could not be done in this study.

As a conclusion, we found a high prevalence of occupational asthma in foundry workers and smoking had an additive effect on respiratory symptoms. Encouragement of smoking cessation, occupational health education to reduce the dust exposure, using protective masks during work period, and periodical medical examination are needed to control occupational asthma.

## Figures and Tables

**Table 1:** The distribution of respiratory symptoms among the foundry workers.

Respiratory symptoms	Workers in production departments (*n* = 286)	Workers in other departments (*n* = 61)	Total (*n* = 347)	*P *
Cough	48	4	52 (14.98%)	0.008*
Phlegm	66	5	71 (20.46%)	0.001*
Breathlessness	26	2	28 (8.06%)	0.041*
Chest tightness	13	1	14 (4.03%)	0.154
Wheezing	6	1	7 (2.01%)	0.803

*Statistically significant.

**Table 2:** The number of workers with occupational asthma and airway obstruction (based on FEV_1_).

Working department	OA (%)	Decrease in FEV_1_ (% of predicted)		
		Mild	Moderate	Severe
Production department (*n* = 286)	48 (16.78%)*	5	38	5
Nonproduction department (*n* = 61)	3 (4.91%)	—	3	—

Total (*n* = 347)	51 (14.69%)	5	41	5

OA: occupational asthma; the airway obstruction was classified according to forced expiratory volume in one second (FEV_1_, % of predicted) results and divided into 3 groups as mild (>79%), moderate (60–79%), and severe (40–59%).

*P = 0.001 compared to non-production department.

**Table 3:** The prevalance of respiratory symptoms among the foundry workers according to the use of protective mask.

Parameter	Presence of protective mask (*n* = 178)	Absence of protective mask (*n* = 104)	Total (*n* = 286)	*P *
Respiratory symptoms (any one or more)	44 (24.7%)	38 (36.5%)	82	0.039
Occupational asthma	19 (10.6%)	29 (27.8%)	48	0.001

**Table 4:** The distribution of occupational asthma and mean FEV_1_ (%) in foundry workers according to dust exposure.

Exposure	Department	Mean respirable dust concentration	OA (*n* = 51)	Mean FEV_1_ (% of predicted)^a^
High-exposure groups	Fettling, cleaning castings (*n* = 64)	10.448 mg/m^3^	15 (23.4%)^b,∗^	87.8
	Core making, furnace (*n* = 53)	10.122 mg/m^3^	14 (26.4%)^c,∗^	89.5
Low-exposure groups	Moulding, melting, and pouring (*n* = 83)	0.322 mg/m^3^	11 (13.2%)^d^	94.2
	After processing (*n* = 86)	0.216 mg/m^3^	8 (9.3%)^e^	91.8
Unexposed group	Nonproduction (*n* = 61)	Non	3 (4.9%)	93.1

OA: occupational asthma, FEV_1_: forced expiratory volume in one second; ^a^the mean FEV_1_ (% of predicted) results of the workers were not found to be different compared to unexposed group (*P* > 0.05). ^b^ *P*: 0.002, ^c^ *P*: 0.001, ^d^ *P*: 0.294, ^e^ *P*: 0.072 compared to unexposed group; *statistically significant.

## References

[1] Schwartz DA, Peterson MW (1998). Occupational lung disease. *Disease*.

[2] Ahn Y-S, Won J-U, Park RM (2010). Cancer morbidity of foundry workers in Korea. *Journal of Korean Medical Science*.

[3] Hahn R, Beck B (1986). Prevalence of chronic bronchitis among foundries’ workers. *Zeitschrift fur Erkrankungen der Atmungsorgane*.

[4] Liss GM, Bernstein DI, Moller DR, Gallagher JS, Stephenson RL, Bernstein IL (1988). Pulmonary and immunologic evaluation of foundry workers exposed to methylene diphenyldiisocyanate (MDI). *Journal of Allergy and Clinical Immunology*.

[5] Löfstedt H, Westberg H, Seldén AI, Bryngelsson I-L, Svartengren M (2011). Respiratory symptoms and lung function in foundry workers using the Hot Box method: a 4-year follow-up. *Journal of Occupational and Environmental Medicine*.

[6] Johnson A, Moira CY, MacLean L (1985). Respiratory abnormalities among workers in an iron and steel foundry. *British Journal of Industrial Medicine*.

[7] Baur X, Bakehe P, Vellguth H (2012). Bronchial asthma and COPD due to irritants in the workplace an evidence based approach. *Journal of Occupational Medicine and Toxicology*.

[8] Global Strategy for Asthma Management and Prevention Global Initiative for Asthma (GINA). http://www.ginasthma.org/local/uploads/files/GINA_Report_March13.pdf.

[9] Chan-Yeung M (1995). Assessment of asthma in the workplace. *Chest*.

[10] Brandli O, Schindler C, Leuenberger PH (2000). Re-estimated equations for 5th percentiles of lung function variables. *Thorax*.

[11] Burge PS, O’Brien IM, Harries MG (1979). Peak flow rate records in the diagnosis of occupational asthma due to isocyanates. *Thorax*.

[12] Ahn YS, Song JS, Kang SK, Chung HK (2000). Understanding the occurrence of lung cancer in foundry workers through health insurance data. *Korean Journal of Preventive Medicine*.

[13] Ahn YS, Korea Occupational Safety Health Agency (2005). Respiratory diseases in foundry workers. *Training Materials for Occupational Respiratory Diseases*.

[14] Cartier A (1994). Definition and diagnosis of occupational asthma. *European Respiratory Journal*.

[15] Banks DE, Tarlo SM, Masri F, Rando RJ, Weissman DN (1996). Bronchoprovocation tests in the diagnosis of isocyanate-induced asthma. *Chest*.

[16] Pellegrino R, Viegi G, Brusasco V (2005). Interpretative strategies for lung function tests. *European Respiratory Journal*.

[17] Orman A, Ellidokuz H, Esme H, Unlu M, Ay A (2004). Evaluation of pulmonary system symptoms with pulmonary functional tests in workers in pulp and paper industry. *Respiratory Diseases*.

[18] Mehta AJ, Henneberger PK, Torén K, Olin A-C (2005). Airflow limitation and changes in pulmonary function among bleachery workes. *European Respiratory Journal*.

